# Substance Misuse Risk and Relapse Prevention Provision Within the Inpatient Forensic Psychiatric Setting in KMMH: A Service Evaluation Project

**DOI:** 10.1192/bjo.2026.11524

**Published:** 2026-06-30

**Authors:** Moustafa Abdelkader, Faizaan Syed, Francis Felix, Karan Veer Singh, Rafey Faruqui

**Affiliations:** Kent and Medway Mental Health NHS Trust, Maidstone, United Kingdom

## Abstract

**Aims:**

Substance and alcohol misuse are highly prevalent and frequently comorbidin forensic psychiatric populations. This comorbidity is clinically important because it is associated with relapse, poorer engagement, and heightened risk of re-offending.

To evaluate substance and alcohol misuse risk/relapse-prevention provision within KMMH inpatient forensic services by: (1) describing the cohort’s substance/alcohol misuse profile and related risks; (2) assessing whether substance/alcohol misuse was assessed at admission; and (3) describing programme offer and uptake.

**Methods:**

Service evaluation using routinely collected clinical data for current inpatients across KMMH forensic services. Data were extracted from electronic health records and analysed descriptively.

**Results:**

Sample: N=102. Gender: male 87/102 (85.3%), female 15/102 (14.7%). Assessment at admission: substance and alcohol misuse assessed in 54/102 (52.9%) and not assessed in 48/102 (47.1%). Active substance misuse was recorded in 14/102 (13.7%) and active alcohol use in 13/102 (12.7%). Withdrawal symptoms/incidents were recorded in 2/101 (2.0%) and detoxification was required in 1/101 (1.0%). IV drug users 3/102 (2.9%). Incidents of ongoing substance misuse: yes 5/102 (4.9%).

Substance categories recorded (multi-label; percentages use N as denominator): Cannabis/cannabinoids 50/102 (49.0%); Stimulants 42/102 (41.2%); Alcohol 32/102 (31.4%); Novel psychoactive substances (NPS) 14/102 (13.7%); Opioids 11/102 (10.8%); Hallucinogens/dissociatives 8/102 (7.8%); Sedatives/hypnotics 4/102 (3.9%).

Comorbid mental health diagnosis (multi-label; percentages use N as denominator): Psychotic illness 64/102 (62.7%); Mood disorder 14/102 (13.7%); Anxiety disorder (9.8%); Personality disorder 24/102 (23.5%); Neurodivergent 22/102 (21.6%); Paraphilias 2/102 (2.0%); Other 7/102 (6.9%).

Substance misuse programme provision: a substance misuse programme was offered to 44/102 (43.1%). Of those offered (n=44), 33/44 (75.0%) attended and 11/44 (25.0%) did not attend.

Previous engagement with substance misuse services (as recorded in the notes–percentages using N as denominator): Community setting 14/102 (13.7%); Acute inpatient setting 3/102 (2.9%); Forensic inpatient setting 16/102 (15.7%); Prison setting 7/102 (6.9%).

**Conclusion:**

This service evaluation highlights two key quality-improvement opportunities. First, nearly half of patients had no recorded drug or alcohol misuse assessment at admission, suggesting inconsistent screening and/or documentation.

Second, although recorded active use and acute withdrawal/detoxification needs were relatively uncommon, a large proportion of the cohort had histories involving multiple substance classes. This supports the case for proactive relapse-prevention pathways in forensic settings, even when current dependence indicators are not prominent.

Attendance was high among those offered a programme (75%), indicating acceptability; however, fewer than half of the overall cohort were documented as being offered a programme, suggesting possible unmet need or variation in eligibility identification.

